# Gut related inflammation and cardiorespiratory fitness in patients with CAD and type 2 diabetes: a sub-study of a randomized controlled trial on exercise training

**DOI:** 10.1186/s13098-021-00655-2

**Published:** 2021-04-01

**Authors:** Susanne Kristine Aune, Rune Byrkjeland, Svein Solheim, Harald Arnesen, Marius Trøseid, Ayodeji Awoyemi, Ingebjørg Seljeflot, Ragnhild Helseth

**Affiliations:** 1 Center for Clinical Heart Research, Department of Cardiology, Oslo University Hospital Ullevål, Ullevål Pb 4956 Nydalen, 0424 Oslo, Norway; 2 Faculty of Medicine, University of Oslo, Oslo, Norway; 3 Department of Cardiology, Oslo University Hospital Ullevål, Oslo, Norway; 4 Section of Clinical Immunology and Infectious Diseases, Oslo University Hospital Rikshospitalet, Oslo, Norway

**Keywords:** Coronary artery disease, Type 2 diabetes, Cardiovascular fitness, Gut leakage, Inflammation, Exercise intervention

## Abstract

**Aim:**

Gut leakage has been shown to associate with low-grade inflammation and lower cardiorespiratory fitness in diabetic subjects. We aimed to investigate whether gut leakage markers related to cardiorespiratory fitness in patients with both coronary artery disease and type 2 diabetes, and whether these were affected by long-term exercise training.

**Methods:**

Patients with angiographically verified coronary artery disease and type 2 diabetes mellitus (n = 137) were randomized to either 12 months exercise intervention or conventional follow-up. A cardiopulmonary exercise test and fasting blood samples were obtained before and after intervention to assess VO_2_peak and the biomarkers soluble CD14, lipopolysaccharide-binding protein and intestinal fatty-acid binding protein as markers of gut leakage.

**Results:**

114 patients completed the intervention satisfactory. VO_2_peak correlated inversely to sCD14 (r = − 0.248, p = 0.004) at baseline. Dividing sCD14 into quartiles (Q), VO_2_peak was significantly higher in Q1 vs. Q2–4 (p = 0.001), and patients in Q2-4 (sCD14 > 1300 ng/mL) had an OR of 2.9 (95% CI 1.2–7.0) of having VO_2_peak below median (< 23.8 ml/kg/min) at baseline. There were no statistically significant differences in changes in gut leakage markers between the two randomized groups (all p > 0.05) after 12 months.

**Conclusions:**

Cardiorespiratory fitness related inversely to sCD14, suggesting physical capacity to be associated with gut leakage in patients with CAD and T2DM. Long-term exercise training did not affect circulating gut leakage markers in our population.

*Trial registration* NCT01232608, Registered 02 November 2010—Retrospectively registered at https://clinicaltrials.gov/ct2/show/NCT01232608?term=NCT01232608&draw=2&rank=1

**Supplementary Information:**

The online version contains supplementary material available at 10.1186/s13098-021-00655-2.

## Introduction

Intestinal dysbiosis and *gut leakage* is hypothesized to contribute to the chronic low-grade inflammatory state associated with obesity, insulin resistance and atherosclerotic cardiovascular disease (CVD) [1, 2]. Gut leakage describes the translocation of bacterial wall products, mainly lipopolysaccharide (LPS), across the gut barrier by either para- or transcellular mechanisms, or by enterocyte damage with subsequent leakage of the intracellular intestinal fatty acid-binding protein (I-FABP) into the circulation [3]. LPS in the circulation interacts with LPS-binding protein (LBP), membrane bound or soluble CD14 (sCD14), activating the toll-like receptor 4 (TLR4) and initiating downstream inflammatory signaling [4]. Elevated levels of LBP and sCD14 have been associated not only with insulin resistance and obesity [5], but also with coronary artery disease (CAD) and all-cause mortality [6, 7]. As levels of LBP and sCD14 have been shown to correlate with LPS, they are acknowledged surrogate markers for endotoxemia [8]. However, an increase in sCD14 is not restricted to LPS exposure, but can be regarded as a marker of monocyte activation [9]. I-FABP is regarded as a marker of intestinal injury or ischemia [10].

Regular physical exercise is recommended by the European society of Cardiology for prevention of cardiovascular events and all-cause mortality, and is associated with a beneficial cardio-metabolic profile and decrease in markers of systemic inflammation [11]. Furthermore, a combination of aerobic and resistance training has been shown to improve insulin sensitivity and glycemic control in subjects with T2DM [12]. Exercise has also been hypothesized to contribute to increased gut microbial diversity, and thus a less leaky gut [13]. In healthy subjects, microbial diversity has been shown to associate with cardiorespiratory fitness [14], whereas in diabetic and pre-diabetic subjects gut leakage markers have been reported elevated, and importantly, modifiable by exercise [15]. However, the link between physical fitness and effects of exercise training on markers of gut leakage has not been extensively studied, particularly in patients with CAD. Our aims were therefore to explore the relationship between cardiorespiratory fitness and gut leakage markers in patients with combined CAD and T2DM. We hypothesized that less aerobically fit patients have higher levels of gut leakage markers than fit patients. Secondly, we aimed to investigate whether long-term exercise can impact circulating gut leakage markers in patients with T2DM and CAD. Our hypothesis was that exercise would reduce gut leakage.

## Methods

### Study population

The present investigation is a sub-study of the “Exercise training in patients with coronary artery disease and type 2 diabetes” (EXCADI) study [16]. Patients with combined T2DM and angiographically verified CAD (n = 137) were included at Department of Cardiology, Oslo University Hospital, Oslo, Norway, between August 2010 and March 2012. The study design has previously been described in detail, and the methods description partly reproduces their wording [16]. In brief, patients were randomized 1:1 to a combination of strength and endurance training with an instructor or to a control group with conventional follow-up by their general practitioner. Patients in the control group were not discouraged to exercise. Diabetic microvascular complications were defined as a history of nephron-, neuro- or retinopathy, and/or abnormal monofilament test and/or (micro-) albuminuria. Patients with previous myocardial infarction (MI) and/or diabetic complications were defined to have advanced vascular disease. Hypertension (HT) was defined as use of antihypertensive medication.

Exclusion criteria were presence of proliferative retinopathy, end-stage renal disease, cancer, stroke or acute MI within the last 3 months, unstable angina, decompensated heart failure, serious arrhythmia, severe valvular disease, severe rheumatologic disease, chronic obstructive pulmonary disease stadium GOLD IV, thromboembolic disease, ongoing infections, severe musculoskeletal disorders, or other disabilities limiting the ability for physical activity.

### Cardiopulmonary exercise test

A cardiopulmonary exercise test using a modified Balke protocol was performed on treadmill before the intervention and approximately one week after the last exercise session [17]. The procedure has previously been described [18]. It was continued until exhaustion or until ended by the physician for safety reasons. Gas exchange was continuously measured by breathing into a Hans Rudolph two-way breathing mask (2700 series; Hans Rudolph Inc, Kansas City, USA), connected to a metabolic cart (Vmax SensorMedics, Yorba Linda, USA) assessing ventilation, oxygen and carbon dioxide content of expired air. VO_2peak_ was defined as the highest average oxygen uptake of consecutive 30 s during the test. The achieved VO_2peak_ was also included if the test had to be terminated because of functional limitations. Maximal respiratory exchange ratio (RER) was measured. The anaerobic threshold (AT) was estimated by the ventilatory equivalent method [19].

### Physical exercise intervention

The exercise group underwent a 12-month training intervention. The physical exercise program was developed and conducted in collaboration with the Norwegian School of Sport Sciences (NIH). Details on the exercise program have previously been described [16]. In brief, the exercise group participated in a total of 150 min of training per week, divided into supervised group-based training sessions of combined aerobic and resistance training two times a week, with an additional home-based individual exercise once a week. The Borg’s rated perceived exertion (RPE) scale was used to guide exercise intensity [20].

### Laboratory methods

Venous blood samples were drawn in fasting conditions by standard venipuncture between 08:00 and 10:00 a.m. before intake of morning medication at time of inclusion and after 1 year. Routine blood samples including HbA1, insulin, CRP and C-peptide were determined by conventional laboratory methods. Insulin resistance was estimated by the updated homeostatic model assessment indexes for insulin resistance (HOMA2-IR). Glucose and insulin values were entered in the computer model to calculate HOMA2-IR [21].

A biobank was established. Serum was prepared by centrifugation within 1 h at 2500*g* for 10 min, and EDTA plasma was prepared by centrifugation within 1 h at 2700*g* for 20 min at 4 °C, both kept frozen at − 80 °C until analyses. I-FABP was determined in serum, measured by enzyme-linked immunosorbent assay (ELISA) (Hycult Biotech, Uden, the Netherlands). sCD14 and LBP were measured in EDTA plasma by ELISAs (R&D Systems Europe, Abingdon, Oxon, UK and Hycult Biotech, respectively). The inter-assay coefficients of variation (CV) were 17.3%, 10.9% and 8.1% respectively.

### Statistical analysis

Demographic data are given as proportions, mean (± SD) or median (25th and 75th percentiles) depending on the data distribution. Differences between groups at baseline were analyzed by Chi square test for categorical data and Mann–Whitney *U* test or Kruskal–Wallis Test as appropriate for continuous data. Correlation analyses were performed by Spearman’s Rho. A univariate logistic regression model was used to estimate odds ratio for peak VO_2_ according to levels of sCD14, and a multivariate regression analysis was used to adjust for variables known to influence peak VO_2_ (age, sex and BMI). Receiver operating characteristic (ROC) curve was performed to explore any predictive value of I-FABP1 for the presence of microvascular complications. Changes in biomarkers from baseline to end of intervention are given as absolute and relative changes. Within-group changes were analyzed by Wilcoxon Signed Rank test, and differences in changes and relative changes between groups were analyzed by Mann–Whitney *U* test. Statistical calculations were performed using SPSS version 25 (SPSS, Inc., Chicago, IL, USA). *p*-values < 0.05 were considered statistically significant.

## Results

### Patient characteristics at baseline

Baseline characteristics are presented in Table 1. Of the 137 included patients, 123 completed the study. Patients with the lowest adherence to the training intervention (< 40% adherence) were excluded (n = 9) from the per protocol analyses [16]. Thus, 114 patients were analyzed for the

**Table 1:** Baseline characteristics of the total population and according to randomized groups

	All (n = 137)	Exercise (n = 52)	Control (n = 62)
Age (years)^a^	64 [41, 81]	65 [48, 81]	65 [41, 77]
Sex (male) *n*, (%)	115 (83.9)	45 (86.5)	50 (80.6)
BMI^b^ (kg/m^2^)	28.7 (25.6, 31.6)	29.4 (25.6, 31.8)	28.3 (25.4, 31.5)
Duration of diabetes (years)	9 (5, 15)	11 (5, 15)	9 (5, 13)
Previous MI^c^, *n* (%)	62 (45.3)	20 (38.5)	31 (50.0)
Microvascular complications^d^	26 (19)	7 (13.5)	10 (16.1)
Advanced vascular disease^e^, *n* (%)	85 (62.0)	28 (53.8)	40 (64.4)
Hypertension, *n* (%)	100 (73.0)	39 (75.0)	46 (74.2)
Current smokers, *n* (%)	23 (16.8)	9 (17.3)	9 (14.5)
HbA1c (%)	7.4 (6.8, 8.3)	7.4 (6.8, 8.4)	7.3 (6.8, 7.9)
Fasting blood glucose	8.1 (6.9, 9.8)	7.5 (6.7, 9.9)	8.0 (6.8, 9.6)
HOMA2-IR^f^	1.3 (0.7, 2.1)	1.1 (0.7, 1.9)	1.3 (0.7, 2.2)
Total cholesterol (mmol/L)	3.9 (3.4, 4.5)	3.8 (3.4, 4.3)	4 (3.3, 4.7)
Triglycerides (mmol/L)	1.4 (1.0, 1.9)	1.4 (1.1, 1.9)	1.4 (1.0, 1.9)
LDL^g^ cholesterol (mmol/L)	2.0 (1.6, 2.6)	1.8 (1.5, 2.5)	2.1 (1.6, 2.7)
CRP^h^ (mg/L)	2.6 (1.2, 5.0)	2.0 (0.9, 4.9)	2.4 (1.2, 5.0)
Medication			
RAAS inhibitors, *n* (%)	97 (70.8)	34 (65.4)	46 (74.2)
Statins, *n* (%)	128 (93.4)	49 (94.2)	58 (93.5)
Metformin, *n* (%)	101 (73.7)	40 (76.9)	46 (74.2)
Sulfonylureas, *n* (%)	48 (35.0)	23 (44.2)	17 (27.4)
Gliptin, *n* (%)	17 (12.4)	6 (11.5)	11 (17.7)
Insulin, *n* (%)	26 (19.0)	10 (19.2)	12 (19.4)
Antiplatelet medication, *n* (%)	129 (94.2)	47 (90.4)	61 (98.4)

Data are presented as median (25, 75 percentiles) if not stated otherwise

^a^Median [min, max]

^b^Body mass index

^c^Myocardial infarction

^d^Microvascular complications were defined as a history of nephron-, neuro- or retinopathy, and/or abnormal monofilament test and/or (micro-) albuminuria

^e^Advanced vascular disease was defined as patients having previous myocardial infarction and/or diabetic microvascular complications in addition to coronary artery disease

^f^Glucose and insulin values were entered in the computer model to calculate HOMA2-IR

^g^Low-density lipoprotein

^h^C-reactive protein

intervention effect; 52 in the exercise group and 62 in the control group. There were no significant differences between the intervention groups at baseline.

There were no significant differences between the groups in changes in weight, waist circumference, energy intake, percentage intake of main nutrients or diabetes medication during the study period, as previously reported [16]. There was also no significant difference in basal metabolic rate (BMR) between the groups at baseline or significant difference in change between the groups after the intervention (data not shown).

### Gut-related biomarkers and cardiorespiratory fitness at baseline

As outlined in Table 2, the intervention groups were comparable as to physical performance at baseline. During the cardiorespiratory exercise test, one hundred and twenty-seven patients (93%) reached RER > 1.10 and/or Borg scale (6–20) > 17. Levels of the gut leakage markers at baseline are also presented in Table 2. The gut leakage markers did not differ between the two randomized groups at baseline.

**Table 2:** Physical performance and levels of gut leakage markers in all patients, and according to intervention groups at baseline

	All (n = 137)	Exercise (n = 52)	Control (n = 62)	*p-*values
Exercise time (min:sec)	8:23 ± 02:39	8:05 ± 02:23	8:47 ± 2:46	0.153
Maximal RER^a^	1.17 (1.08, 1.23)	1.16 (1.04, 1.23)	1.16 (1.09, 1.23)	0.260
Maximal Borg rating scale (6–20)^a^	17 (17, 19)	17 (17, 18)	18 (17, 19)	0.106
VO_2peak_ (mL/kg/min)	24.7 ± 5.9	24.4 ± 5.3	25.0 ± 6.6	0.556
VO_2peak_ (L/min)	2.17 ± 0.56	2.16 ± 0.52	2.18 ± 0.59	0.868
AT (mL/kg/min)	18.9 ± 4.1	18.9 ± 3.9	19.9 ± 4.6	0.263
AT (L/min)	1.68 ± 0.42	1.69 ± 0.36	1.75 ± 0.47	0.466
sCD14 (ng/mL)^a^	1504 (1299, 1760)	1550 (1334, 1710)	1435 (1276, 1792)	0.456
LBP (µg/mL)^a^	13.1 (10.3, 17.1)	12.7 (9.2, 15.8)	12.7 (10.6, 16.4)	0.562
I-FABP (pg/mL)^a^	988 (783, 1497)	1004 (766, 1561)	971 (844, 1529)	0.891

Data are presented as mean ± SD if not stated otherwise

*p*-values are given for differences between the randomized groups. p-values < 0.05 were considered statistically significant.

*RER* Respiratory exchange rate, *AT* anaerobic threshold, *VO*_*2peak*_ Peak oxygen uptake

^a^Median (25, 75 percentiles)

In the total population, VO_2peak_ was inversely correlated to sCD14 (*p* = 0.004) (Table 3, Additional file 2: Figure S1) and CRP (r = − 0.320, p < 0.001). Otherwise, no significant correlations between markers of gut-related inflammation and variables reflecting physical performance were observed.

**Table 3:** Coefficients of correlation between gut leakage markers and physical fitness at baseline

	VO_2peak_ (ml/kg/min)	AT (ml/kg/min)
sCD14	**r = − 0.248**	r = − 0.132
	**p = 0.004**	p = 0.172
LBP	r = − 0.070	r = − 0.103
	p = 0.419	p = 0.289
I-FABP	r = − 0.059	r = − 0.001
	p = 0.498	p = 0.993

*AT* anaerobic threshold, *VO*_*2peak*_ Peak oxygen uptake. p-values < 0.05 were considered statistically significant

**Fig. 1:**
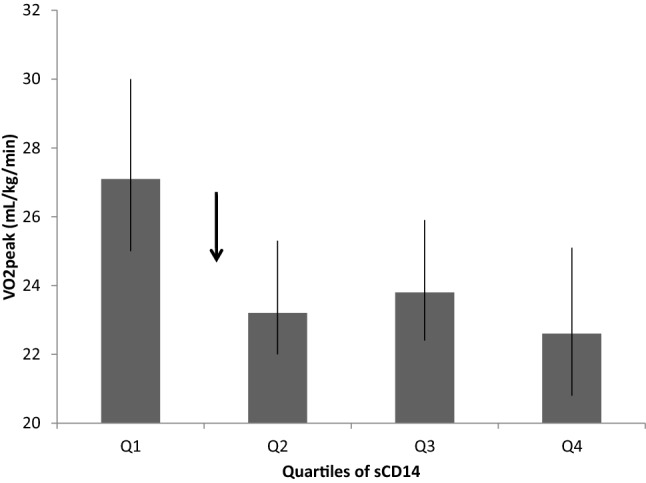
VO_2peak_ in quartiles of sCD14. Q1: < 1300 ng/mL Q2: 1300–1504 ng/mL Q3: 1504–1759 ng/mL Q4: > 1759 ng/mL (error bars indicate 25th, 75th percentiles). Arrow indicates cut off level

**Fig. 2:**
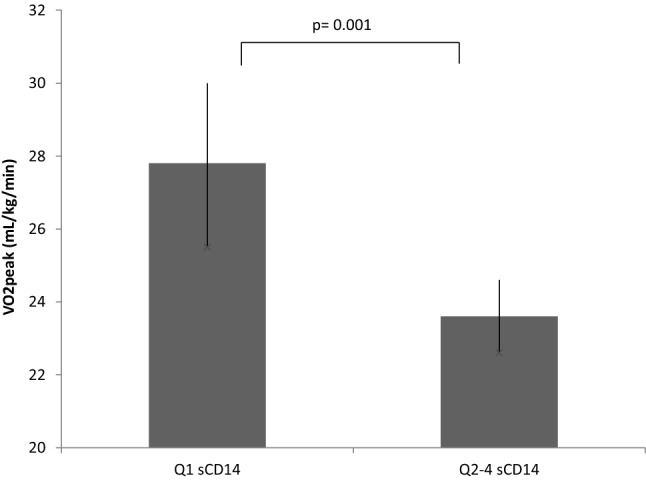
VO_2peak_ in dichotomized groups of sCD14 (error bars indicate 25th, 75th percentiles). *p*-value is given for difference between groups

VO_2peak_ levels differed significantly between quartiles of sCD14 (*p* = 0.005) (Fig. 1). Based on a visual cut-off between the lowest quartile (Q_1_) and the three highest quartiles (Q_2–4_) (Fig. 1, arrow), sCD14 was dichotomized into Q_1_ and Q_2–4_ for further analyses. VO_2peak_ was significantly lower in Q_2–4_ compared to Q_1_ (*p* = 0.001) (Fig. 2). Patients in Q_2–4_ (sCD14 > 1300 ng/mL) had an odds ratio (OR) of 3.2 (95% CI 1.4–7.5) of having a VO_2peak_ below median (< 23.8 mL/kg/min). The results were still significant after adjusting for age, sex and BMI, with an OR of 2.9 (95% CI 1.2–7.0).

### Markers of gut leakage, glucometabolic state and diabetic complications

Levels of sCD14 at baseline correlated significantly to fasting blood glucose (r = 0.174, *p* = 0.044), but not to HbA1c, HOMA2-IR and diabetes duration (Additional file 1: Table S1). LBP correlated significantly to waist circumference (r = 0.198, *p* = 0.023), but not to any glucometabolic variable. Patients with microvascular complications had significantly higher I-FABP than patients without these diabetic complications (*p* = 0.010), with an AUC of 0.66 (95% CI 0.56, 0.77) in ROC curve analysis (*p* = 0.010).

### Effect of exercise on markers of gut leakage

**Fig. 3:**
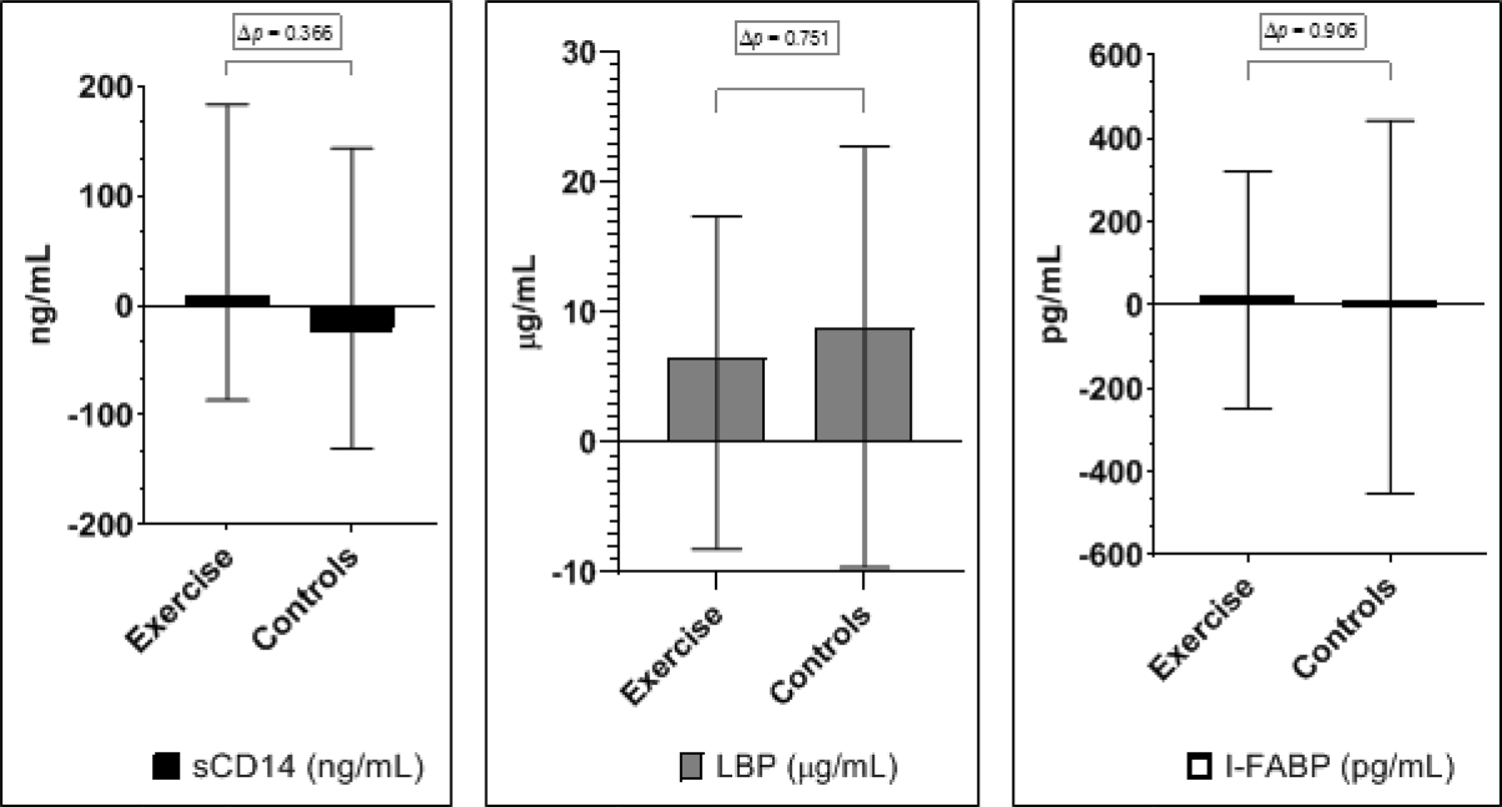
Median changes in gut leakage markers from baseline to 12 months in the randomized groups in absolute values (error bars indicate 25th, 75th percentiles). ∆p-values refer to difference in change between groups

**Fig. 4:**
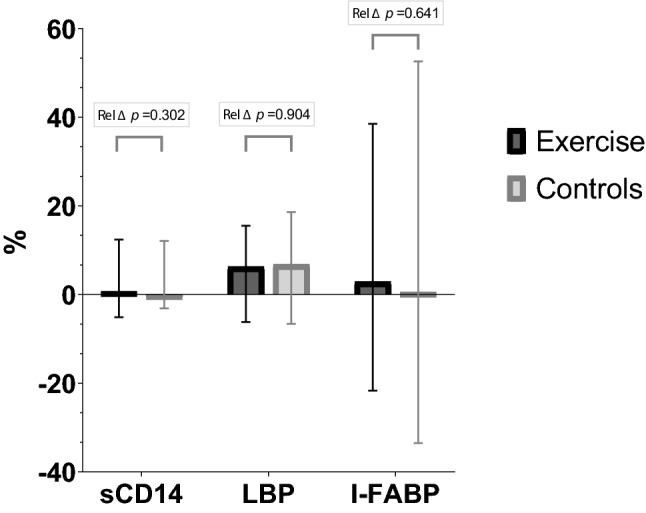
Relative changes in gut leakage markers from baseline to 12 months in the randomized groups. The relative changes are presented as median (error bars indicate 25th, 75th percentiles). Rel ∆*p*-values refer to difference in change between groups

Changes in markers of gut leakage according to the intervention groups from baseline to 12 months are illustrated in Fig. 3. Relative changes during the intervention are presented in Fig. 4. As previously reported, there was a non-significant increase in mean VO_2peak_ in the exercise group of 0.8 mL/kg/min (95% CI − 0.2 to 1.8; *p* = 0.107), and the difference in change from baseline to 12 months was not significant between the exercise group and the control group [16]. The exercise intervention did not significantly affect the gut leakage markers after 12 months, neither in absolute nor in relative numbers (Figs. 3 and 4). The same lack of intervention effect was observed for patients without advanced vascular disease (n = 46). For patients in the intervention group who increased their VO_2peak_ numerically during the intervention period (n = 28), no significant changes in gut leakage markers were observed (data not shown).

There were no significant within group changes from baseline to 12 months in any of the gut leakage markers, neither in the exercise nor the control group (data not shown).

## Discussion

This study aimed to explore the relationship between cardiorespiratory fitness and markers of gut leakage in patients with combined T2DM and CAD. Our main finding was a significant inverse association between VO_2peak_ and levels of sCD14 and CRP, indicating increased monocyte activation and inflammation, and possibly increased gut leakage, in individuals with poorer cardiorespiratory fitness. We did not, however, find any significant association with LBP, which is a more robust marker of gut leakage. There was no significant effect of 12 months exercise intervention on levels of gut leakage markers. This is, to the best of our knowledge, the first report on the relationship between cardiorespiratory fitness and gut leakage markers in patients with combined T2DM and CAD.

Although we did not measure the gut microbial composition or LPS levels per se, LPS is a potent stimulator of sCD14 release [22]. Our result showing low cardiorespiratory fitness to be associated with high levels of sCD14 could indicate a relationship to a dysbiotic and leaky gut. sCD14 has, however, been shown to be released upon interaction with other TLR ligands and inflammatory cytokines as well [9], and could therefore be considered a marker of monocyte activation and inflammation in general, not necessarily restricted to LPS and TLR4 interaction. The observed similar inverse relationship between CRP and VO_2peak_ in our patients supports the theory of a general inflammatory process.

The underlying mechanisms by which a dysbiotic intestinal microbiota, gut leakage and cardiorespiratory fitness interact remain vastly unexplained. Our cross-sectional study of the baseline association limits our possibility of suggesting any causality, but previous studies link both intestinal dysbiosis and low cardiorespiratory fitness to a state of low-grade inflammation [2, 23]. Chronic inflammation is also present in obesity [24], but our findings were significant even after adjusting for BMI. Collectively, these results allow us to speculate that change in gut permeability and subsequent gut leakage is linked to the activation of inflammatory signaling pathways, being relevant in T2DM, atherosclerosis and cardiorespiratory fitness. However, one cannot rule out the possibility that patients with higher inflammation have greater cytokine-induced fatigue and are less likely to exercise, and therefore have lower baseline VO_2peak_ and higher sCD14.

Beyond significant higher levels of I-FABP in patients with microvascular complications, the investigated gut leakage markers did not correlate to glucose control or any marker of glucometabolic state, except for a significant association between sCD14 and fasting blood glucose (*p* = 0.044). The significant correlation found between LBP and waist circumference, is in line with previous studies reporting LBP to be elevated in obesity and in patients with an unhealthy adipose tissue distribution [25].

### Effect of exercise training

There were no significant changes in any of the gut leakage markers after 12 months of combined resistance and endurance training. We have previously reported that the exercise group overall did not improve their VO_2peak_, whereas patients without advanced vascular disease did [16]. As we found sCD14 linked to cardiorespiratory fitness, we hypothesized that levels of sCD14 would be reduced in those who increased their VO_2peak_ during the exercise period, but no such relationship was found. It is possible that this group of patients, with both CAD and several years of diabetes with various macro- and microvascular complications, had too advanced disease to reach the presumed effect on reduction in gut leakage markers after 12 months. We could, however, not find any effect of exercise on the gut leakage markers when analyzing patients without advanced vascular disease separately.

The lack of intervention effect in the current study could also be due to lack of power, as power calculations were not specifically performed for the present hypothesis. It may also have been affected by the extent of exercise adherence, which was quite low, with an average of 67% (55%, 83%) [16]. However, it is unclear how much exercise is needed for patients to benefit from it, although we do know that 150/min per week is recommended on a general basis for patients with T2DM [26], and supervised combined resistance and endurance training has been shown to be the best [27].

Our patients were all on medication, of which several are known to have immunomodulary effects to some extent. Almost 74% of the patients were on metformin that has been shown to have a variety of immunomodulatory effects [28]. Although the mechanisms of action and clinical relevance are unclear, and we found no significant difference between patients on and without metformin (data not shown), it is possible that any exercise effect on levels of gut leakage and inflammation, could be masked by metformin usage. In addition, over 90% were on aspirin, known to have anti-inflammatory effects [29], and 35% were on sulfonylureas, which also seem to possess anti-inflammatory properties [30]. This might have modulated the inflammatory response to LPS exposure in our patients, and could have attenuated the measurable effect of the exercise intervention.

### Limitations

The main limitation of our study is that it was initially designed for the purpose of studying the effect of exercise training on HbA1c [16], thus not for considering effects on the investigated gut leakage markers. Although patients with ongoing infections were excluded from the study at baseline, there was no exclusion of patients who had used antibiotic treatment in the months prior to inclusion. Other studies have excluded patients using antibiotics up to 6 months prior to inclusion [14]. We have not measured levels of LPS, which may have added to the results. However, LPS is unstable in the circulation, and the most used laboratory method for LPS quantification, the limulus amebocytes lysate assay, is encumbered with limitations. We also did not analyze the interventional effects on the microbiota itself. Finally, although we know that different diets alter the gut microbiota differently, we did not control the participants’ diet in details during the intervention [31].

## Conclusion

The inverse association demonstrated between VO_2peak_ and levels of sCD14 and CRP, suggests a significant interaction between cardiorespiratory fitness and gut-related inflammation in our patients with both type 2 diabetes and coronary artery disease.

A 12 months exercise intervention program did, however, not affect gut leakage markers in this patient group.

## Supplementary Information

**Additional file 1**: **Table S1.** Baseline correlations between markers of gut leakage and glucometabolic state are presented in this table.

**Additional file 2**: **Figure S1.** Correlation plot between VO_2_peak and sCD14 at baseline.

## Data Availability

The datasets used and analyzed during the current study are available from the corresponding author on reasonable request.

## Abbreviations

CAD: Coronary artery disease
CVD: Cardiovascular disease
T2DM: Type 2 diabetes mellitus
VO_2peak_: Peak oxygen uptake
sCD14: Soluble cluster of differentiation 14
LPS: Lipopolysaccharide
LBP: LPS-binding protein
I-FABP: Intestinal fatty-acid binding protein
TLR4: Toll-like receptor 4
GOLD: Global initiative for chronic obstructive lung disease
MI: Myocardial infarction
AT: Anaerobic threshold
RER: Respiratory exchange ratio
RPE: Rated perceived exertion
ELISA: Enzyme-linked immune sorbent assay
HOMA2-IR: Updated homeostatic model assessment of insulin resistance
SCFA: Short chain fatty acid
EXCADI: Exercise training in patients with coronary heart disease and Type 2 diabetes
