# Supplementary material for: Gut related inflammation and cardiorespiratory fitness in patients with CAD and type 2 diabetes: a sub-study of a randomized controlled trial on exercise training
Source: Diabetol Metab Syndr. 2021 Apr 1;13:36. doi: 10.1186/s13098-021-00655-2 (PMC8017653; doi:10.1186/s13098-021-00655-2)
Supplement: Supplementary file 1 — Additional file 1: Table S1. Baseline correlations between markers of gut leakage and glucometabolic state are presented in this table. [file 13098_2021_655_MOESM1_ESM.pdf]

Supplementary table 1.

	sCD14	LBP	I-FABP
BMI[1] (kg/m^2)	r=0.017	r=0.156	r=0.028
	p=0.841	p=0.071	p=0.742
Waist circumference (cm)	r=0.109	**r=0.198**	r=0.124
	p=0.212	**p=0.023**	p=0.154
Years of diabetes (years)	r=0.066	r=-0.052	r=0.117
	p=0.453	p=0.553	p=0.117
Glucose (mmol/L)	**r=0.174**	r=0.123	r=0.031
	**p=0.044**	p=0.156	p=0.723
HOMA2-IR[2]	r=-0.011	r=0.123	r=0.049
	p=0.901	p=0.173	p=0.590
HbA1c (%)	r=0.088	r=0.061	r=0.049
	p=0.308	p=0.478	p=0.605

Baseline correlations between markers of gut leakage and glucometabolic state are presented in this table. [1]BMI; body mass index, [2]HOMA2-IR; homeostatic model assessment for insulin resistance.
