# Supplementary material for: Gut related inflammation and cardiorespiratory fitness in patients with CAD and type 2 diabetes: a sub-study of a randomized controlled trial on exercise training
Source: Diabetol Metab Syndr. 2021 Apr 1;13:36. doi: 10.1186/s13098-021-00655-2 (PMC8017653; doi:10.1186/s13098-021-00655-2)
Supplement: Supplementary file 2 — Additional file 2: Figure S1. Correlation plot between VO2peak and sCD14 at baseline. [file 13098_2021_655_MOESM2_ESM.pdf]

Supplementary Figure 1 Correlation plot between $VO2_{peak}$ and sCD14 at baseline.

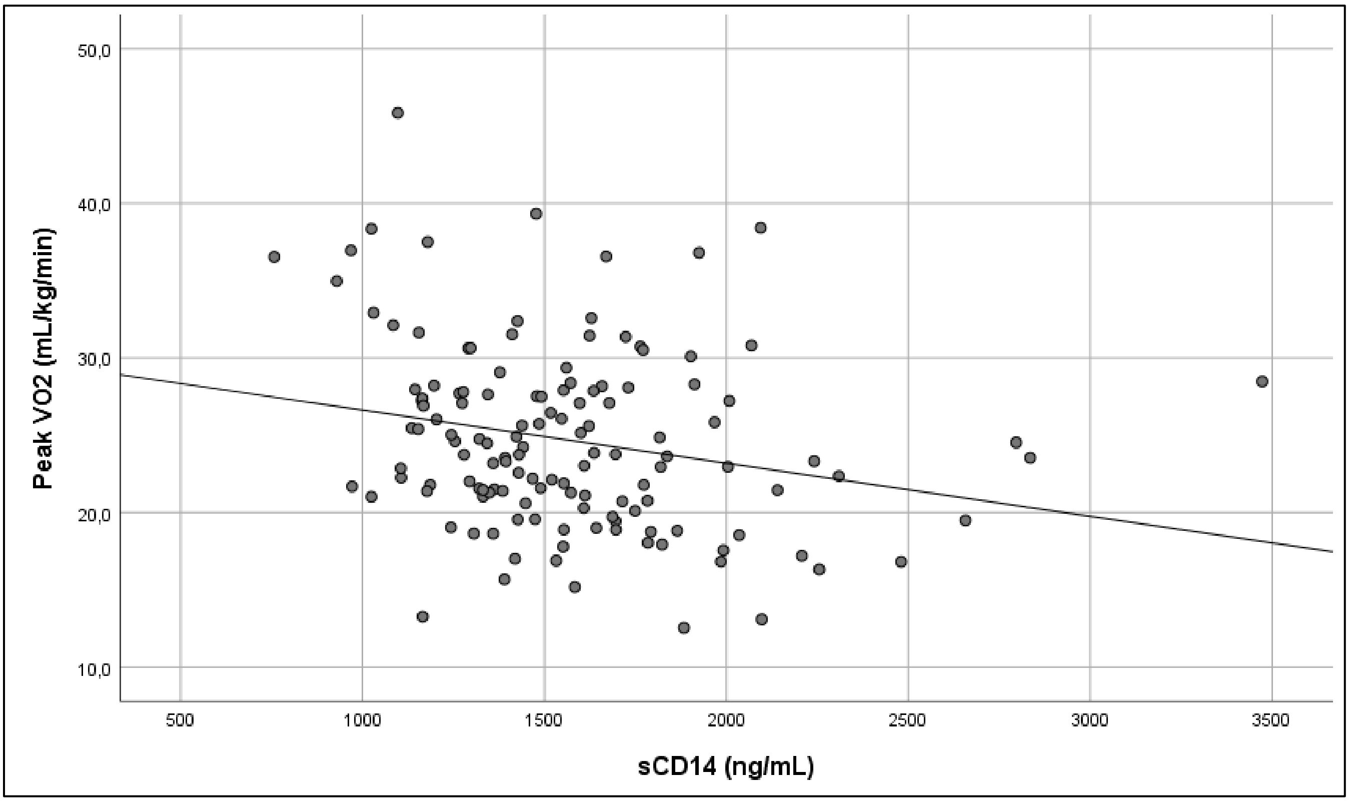

The line represents the best fitted line.
